# Psychometric Properties of the Death Anxiety Beliefs and Behaviors Scale: A Methodological Study in Türkiye

**DOI:** 10.1007/s10943-026-02703-5

**Published:** 2026-06-21

**Authors:** Esra Yıldız, Zeynep Öztürk, Gülcan Bahçecioğlu Turan

**Affiliations:** 1 Faculty of Nursing Atatürk Üniversitesi, Erzurum, Turkey; 2 Department of Nursing, Faculty of Health Sciences, Erzurum Teknik University, Erzurum, Turkey; 3 Department of Nursing, Faculty of Health Sciences, Fırat University, Elazığ, Turkey

**Keywords:** Validity, Reliability, Death anxiety beliefs and behaviors scale, Türkiye, Nursing, Spiritual health

## Abstract

Death anxiety is a multidimensional construct encompassing emotional, cognitive, and behavioral responses to mortality. In societies like Türkiye, where cultural and spiritual values significantly shape the perception of death, valid measurement tools are essential for understanding how individuals experience these concerns. The Death Anxiety Beliefs and Behaviors Scale (DABBS) is a comprehensive instrument capturing these dimensions; however, no validated Turkish version has been available. This study aimed to adapt the DABBS into Turkish and evaluate its structural validity and reliability among adults in Türkiye. A methodological and cross-sectional study design was employed with data collected from 195 adults across different regions of Türkiye. Participants completed an online survey including a Descriptive Form and the 18-item Turkish version of the scale (DABBS-T). Translation and cultural adaptation followed international guidelines. Construct validity was assessed through confirmatory factor analysis (CFA), and reliability was evaluated using Cronbach’s alpha and test–retest stability. CFA supported the original three-factor structure of the scale—emotion, belief, and behavior—with factor loadings ranging from 0.530 to 0.940. The model demonstrated acceptable fit indices (*χ*^2^/d*f* = 2.00, RMSEA = 0.072, CFI = 0.97, TLI = 0.97). Internal consistency was high across all subscales (Cronbach’s alpha = 0.849–0.932). Test–retest reliability coefficients ranged from 0.903 to 0.947, indicating strong temporal stability. No item removal or structural modification was required, suggesting that the Turkish version retained the theoretical integrity of the original instrument. The DABBS-T is a valid and reliable tool for assessing death-related beliefs and behaviors among Turkish adults. Given the scale’s strong psychometric performance, it is highly suitable for research and clinical settings, particularly in exploring the intersection of spiritual beliefs and psychological health. It facilitates cross-cultural comparisons and contributes to a broader understanding of death anxiety within diverse cultural frameworks.

## Introduction

Death, the inevitable end of all humanity, affects the psychological state of individuals. Death anxiety is defined as the fear, anxiety, or discomfort caused by the thought of one’s own death or the death of a loved one (Menzies et al., 2018; Menzies et al., 2022). This anxiety affects both the psychology of individuals and their health-related behaviors, religious beliefs and lifestyle choices (Yıldırım & Kocatepe, 2021; Zuccala et al., 2022). Particularly in collectivist and faith-based societies, death is not merely a biological end but a transition shaped by spiritual beliefs, which directly influences how anxiety is manifested and managed. The effects of death anxiety on health and lifestyle make it essential to evaluate death anxiety. (Cai et al., 2017; Menzies et al., 2022).

Scales developed to measure death anxiety provide both assessment of death anxiety and guidance in planning strategies to cope with it. (Cai et al., 2017; Menzies et al., 2022). It is reported that measurement tools that assess beliefs and behaviors related to death anxiety contribute to the literature in evaluating individuals’ death-related situations. (Menzies et al., 2018, 2019, 2022).

### Literature Review

However, cultural adaptation of the scales and conducting validity and reliability studies are critical. Because cultural differences can influence both society’s and individuals’ attitudes and behaviors toward death, assessments must be conducted with sensitivity (Awadalla et al., 2003; Hambleton & Patsula, 1999; Peters et al., 2013). In this process, the psychometric validation and translation of religious and spiritual measures must follow rigorous methodological standards to ensure cultural relevance (Koenig & Al Zaben, 2021). The Death Anxiety Beliefs and Behaviors Scale (DABBS) has emerged in the literature as a comprehensive instrument measuring individuals’ beliefs and behaviors associated with death anxiety (Menzies et al., 2022). Dong et al. conducted the validity and reliability study of this scale in Chinese (Dong et al., 2024). Mazidi et al. conducted the validity and reliability study in Persian with adolescents (Mazidi et al., 2026).

In Turkiye, several scales have been developed to assess death anxiety (Akça & Köse, 2008; Sarıkaya & Baloğlu, 2016). However, there is currently no validated tool in the Turkish literature that specifically assesses the maladaptive beliefs and avoidance behaviors associated with death anxiety. For example to reduce death anxiety—especially in clinical and spiritual care settings—require a deep understanding of underlying beliefs, emotions, and behaviors, it is crucial to introduce tools that capture these parameters within the local context.

In societies such as Türkiye, where Islamic traditions and culturally embedded beliefs about mortality and the afterlife are highly influential, death is not only perceived as a biological endpoint but also as a meaningful spiritual and existential transition. Within this context, death-related cognitions, avoidance behaviors, and coping responses are often shaped—directly or indirectly—by religious and spiritual worldviews. Although the DABBS does not explicitly measure religiosity or spirituality, its underlying constructs (e.g., beliefs about death and avoidance of death-related stimuli) are conceptually intertwined with existential meaning systems that are frequently informed by religious frameworks.

From this perspective, the adaptation of the DABBS is relevant to the scope of a journal focused on religion and health, as it provides a psychometrically sound tool that enables researchers to examine how death-related cognitions interact with religious and spiritual orientations and how these processes may influence psychological and health-related outcomes. Previous studies have consistently shown that religious beliefs and spiritual coping mechanisms shape individuals’ perceptions of mortality, death anxiety, and psychological adjustment in the face of end-of-life concerns (Yıldırım & Kocatepe, 2021; Zuccala et al., 2022). Therefore, validated instruments assessing death-related constructs are essential for advancing empirical research in the religion–health interface (Koenig & Al Zaben, 2021; Koenig & Carey, 2024).

Accordingly, the Turkish adaptation of the DABBS contributes indirectly but meaningfully to this field by providing a tool that can be used in future studies investigating how culturally and religiously shaped belief systems relate to death anxiety and health outcomes. At the same time, it is important to acknowledge the risk of conceptual overlap or “scale contamination,” whereby constructs related to spirituality, cognition, and mental health may become inadvertently conflated, potentially affecting interpretability (Koenig & Carey, 2024).

This study will not only address a gap in the Turkish literature but also enable meaningful cross-cultural comparisons of death-related anxiety and behaviors. Consequently, this study aimed to adapt the Death Anxiety Beliefs and Behaviors Scale (DABBS) into Turkish and to evaluate its structural validity and reliability among a Turkish adult population. By establishing a clean factor structure, this study also aims to provide a tool that allows for analyzing the relationship between spirituality and health without the issues associated with contaminated measures (Koenig & Carey, 2025).

## Materials

### Design of Study

This study was conducted as a methodological and cross-sectional research.

#### Translation and Cultural Adaptation

Permission to translate the scale was obtained from the author via email. The original scale was translated by two independent translators, and a text was obtained by comparing these two translations. The resulting text was then submitted to experts in the field for review to ensure linguistic validity. Finally, a back-translation was performed. Following this, a pilot study was conducted with ten participants to assess the scale’s comprehensibility. This data were not included in the final dataset. After the cognitive assessment was completed, all items were reviewed one last time by the authors to prevent misunderstandings. Based on these suggestions, the scale was finalized (Koenig & Al Zaben, 2021). In translating the DABBS into Turkish, studies on scale contamination were taken into consideration; specifically, the focus was on the content reflecting beliefs, feelings, and avoidance behaviors related to death anxiety. Care was taken to preserve the conceptual content of the original scale during translation and adaptation (Koenig & Carey, 2025).

### Participants

The study was conducted across Turkiye between September 2024 and 2025 dates, with participants completing the research forms online through social media platforms from different provinces.

To conduct factor analysis for scale validity, the sample size must be 5–10 times the number of items in the scale. Therefore, since the DABBS has 18 items, the target sample size was at least 180 participants. A total of 320 participants were reached within the specified time period. The study was terminated with 195 participants, as 87 did not want to participate in the study and 38 did not meet the research criteria (being 18 years or older, and filling out the survey form in 4–5 min).

Completion of the research forms took approximately 10–15 min. To assess temporal stability, the DABBS-T was re-administered to the same group via an online link two weeks later.

#### Used Tools

The study data were collected using the “Descriptive Form” and the “Death Anxiety Beliefs and Behaviors Scale (DABBS).”

*Descriptive Form* This form consisted of six questions regarding age, marital– employment statues, gender, education level, and having of chronic illness.

*Death Anxiety Beliefs and Behaviors Scale (DABBS)* Developed by Menzies et al. (2022) to examine emotions, beliefs, and behaviors related to death anxiety. The scale consists of 18 items and three sub-dimensions, rated on a 5-point Likert scale (1–5). An increase in the score obtained on the scale indicates increased death anxiety. These sub-dimensions and item contents are as follows: *Emotion Subscale* Four items (1, 2, 3, 4) examining emotions related to death anxiety, with scores ranging from 4 to 20. *Belief Subscale* Seven items (5–11) assessing maladaptive beliefs regarding death anxiety, with scores ranging from 7 to 35. *Behavior Subscale* Seven items (12–18) assessing avoidance behaviors related to death anxiety, with scores ranging from 7 to 35 (Menzies et al., 2019).

### Data Analysis

Data were analyzed using SPSS 27 and LISREL software. Descriptive statistics were used to summarize participant characteristics. Item–total correlations and internal consistency coefficients were calculated to evaluate item performance. Prior to analyses, data screening procedures were conducted to ensure data quality. The dataset was examined for missing data, duplicate responses, and outliers, and no problematic cases were identified. As the survey used a forced-response format, no missing data were observed. Normality assumptions were evaluated by examining skewness and kurtosis values, which indicated that the data were suitable for structural equation modeling. Confirmatory factor analysis (CFA) was conducted using LISREL with maximum likelihood (ML) estimation to evaluate the construct validity of the DABBS-T. Exploratory factor analysis (EFA) was not performed, as the study aimed to test a previously established factorial structure of an existing instrument within a cross-cultural adaptation framework. In such studies, CFA is considered appropriate for evaluating predefined measurement models (Beaton et al., 2000; Brown, 2015; Prinsen, et al., 2018). Model fit was assessed using *χ*^2^/d*f*, RMSEA, CFI, TLI, SRMR, and RMR indices. Reliability was evaluated using Cronbach’s alpha, McDonald’s omega coefficients, and intraclass correlation coefficient (ICC). Test–retest reliability was also examined.

### Ethical Considerations

Ethical approval for the study was obtained from the Ethics Committee of Atatürk University Faculty of Medicine (approval no: B.30.2.ATA.0.01.00/413). Before starting the online survey, participants were informed about the study and presented with an “Informed Consent Form.” Those who did not provide consent were not allowed to participate.

Participants were reminded that they had the freedom to withdraw at any stage (“Respect for Autonomy”), and that their personal information would be kept confidential (“Confidentiality and Privacy”). Throughout the study, the principles of the Declaration of Helsinki on the protection of human rights were strictly followed.

## Results

The participants’ ages ranged from 18 to 73 years, with a mean age of 26.93 ± 10.73. It was determined that 69.7% of the participants were female, 77.4% were single, 94.4% had an undergraduate or higher degree, 72.8% were not employed, and 91.8% did not have a chronic illness (Table 1).

**Table 1:** Participant characteristics (*n* = 195)

	Number (*n* = 195)	%
Gender		
Women	136	69.7
Man	59	30.3
Marital statues		
Married	44	22.6
Single	151	77.4
Employment statues		
Yes	53	27.2
No	142	72.8
Chronic illness statues		
Yes	16	8.2
No	179	91.8
Educational statues		
Literate	1	0.5
Primary education	3	1.5
High school	7	3.6
Undergraduate and above	184	94.4
	Mean ± SD	Min–Max
Age (yr)	26.93 ± 10.73	18–73

### Validity

In the study, the item-level content validity index (I-CVI) ranged between 0.80 and 1.00, while the DABBS-T-level content validity index (S-CVI) was found to be 0.96.

The KMO value was determined as 0.901. Bartlett’s Test of Sphericity was found to be significant (*χ*^2^ = 2169.440, *p* = 0.000). The total explained variance was found to be 63%. The CFA results indicated that factor loadings ranged between 0.530 and 0.940, and the three-factor structure was confirmed (Table 2).

**Table 2:** DABBS-T items means, item–total correlation coefficients, and CFA factor loadings

DABSS-T items	Mean ± SD	Item–total correlations	Factor loading weigh		
			*F*1	*F*2	*F*3
Item1	2.75. ± 1.03	0.628	0.770		
Item2	2.76 ± 1.12	0.689	0.880		
Item3	2.84 ± 1.06	0.716	0.930		
Item4	2.90. ± 1.07	0.738	0.940		
Item5	2.93 ± 1.02	0.534		0.610	
Item6	2.65 ± 1.19	0.562		0.670	
Item7	2.34 ± 1.06	0.590		0.690	
Item8	3.07 ± 1.14	0.638		0.720	
Item9	3.65 ± 1.11	0.564		0.590	
Item10	2.56 ± 1.10	0.728		0.790	
Item11	3.16 ± 1.13	0.538		0.610	
Item12	2.68 ± 1.00	0.556			0.650
Item13	3.01 ± 1.07	0.584			0.730
Item14	2.15 ± 1.14	0.525			0.540
Item15	3.41 ± 1.18	0.462			0.530
Item16	2.13 ± 1.17	0.529			0.610
Item17	2.69 ± 1.11	0.598			0.780
Item18	2.23 ± 1.11	0.523			0.610

When examined, it was seen that the highest arithmetic mean was item 9 (3.65 ± 1.11) and the lowest arithmetic mean was item 16 (2.13 ± 1.17).

**Fig. 1:**
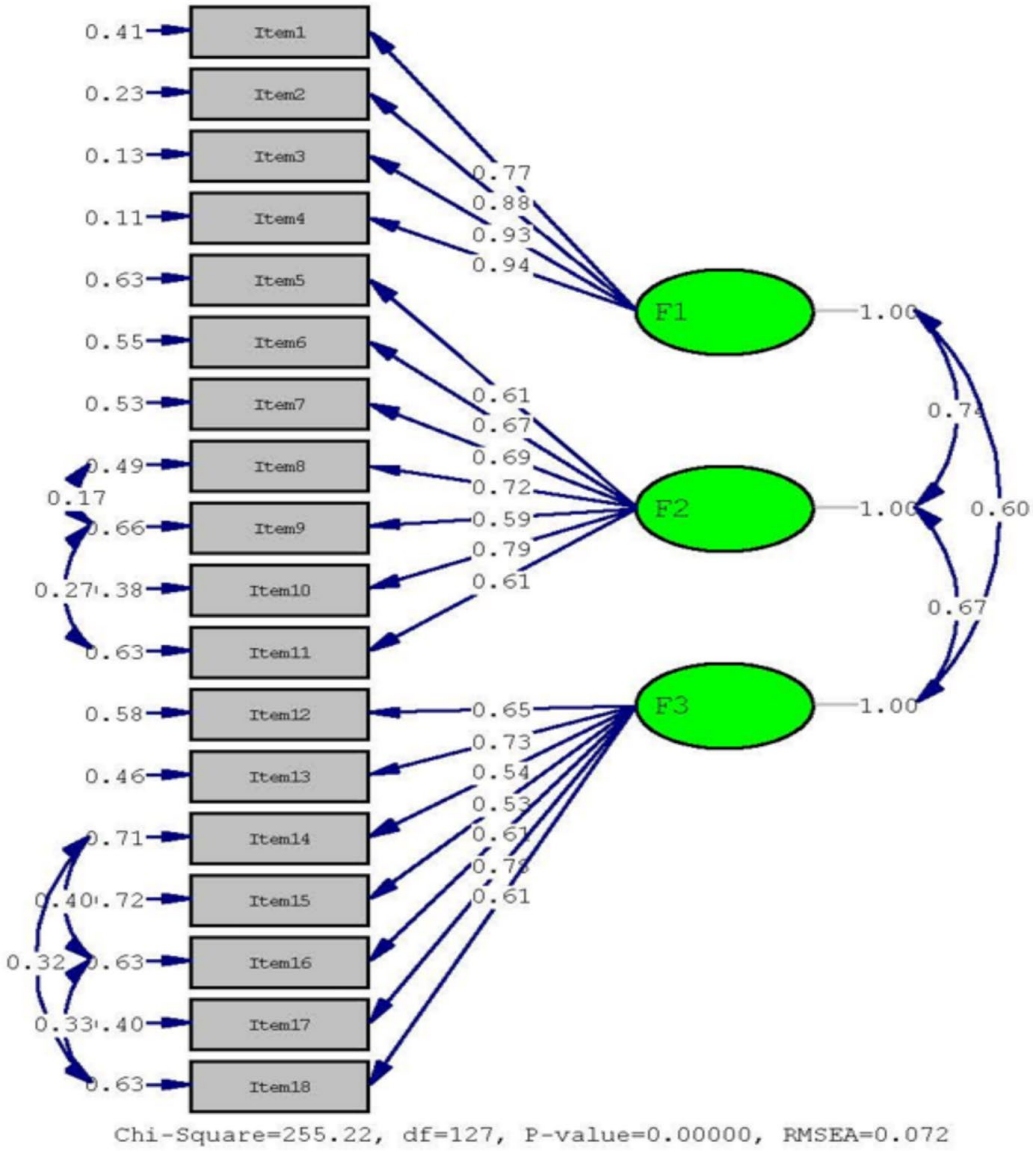
Path diagram regarding the factor structure of the DABBS-T

According to the results of the analysis, the CFA fit index values were obtained as follows: *χ*^2^ = 255.22, d*f* = 127 (*p* < 0.05), *χ*^2^/d*f* = 2.00, RMSEA = 0.072, CFI = 0.97, SRMR = 0.061, TLI = 0.97, RMR = 0.076, and AIC = 343.22 (Table 3). Figure 1 presents the path diagram drawn as a result of CFA.

**Table 3:** Results of CFA of DABSS-T

	Found	Fit criteria
*x*^2^/df	2.00	Acceptable fit
RMSEA	0.072	Acceptable fit
CFI	0.97	Perfect fit
SRMR	0.061	Acceptable fit
RMR	0.076	Acceptable fit
TLI	0.97	Perfect fit
AIC	343.22

An examination of Table 4 reveals a positive, moderate, and statistically significant correlation between the sub-dimensions. Omega values ranged from 0.913 for the total to 0.844–0.935 for the sub-dimensions.

**Table 4:** Correlation values, reliability values

DABBS-T and sub-dimensions	*F*1	*F*2	*F*3	DABSS	*α*	Ω
*F*1	1				0.932	0.935
*F*2	0.643**	1			0.861	0.859
*F*3	0.487**	0.531**	1		0.849	0.844
DABBS	0.806**	0.876**	0.825**	1	0.918	0.913

*F* Factor, *DABSS-T* Death Anxiety Beliefs and Behaviours Scale-Turkish, *F1* Affect, *F2* Beliefs, *F3* Behaviours

^**^p: 0.01 level

### Reliability

The Cronbach’s alpha coefficient of the DABBS-T was calculated. It was found to be 0.932 for the “*F*1” subscale, 0.861 for the “*F*2” subscale, 0.849 for the “*F*3” subscale, and 0.918 for the total scale. In addition, the omega reliability coefficients of the DABBS-T were determined to range between 0.844 and 0.935 (Here Table 4). The item–total correlation values of the DABBS-T were changed to range between 0.462 and 0.738.

To assess the temporal consistency of the DABBS-T, test–retest measurements were administered to 30 participants at 15-day intervals. The observed correlations between the two administrations were found to be strong and statistically significant. Furthermore, comparative analyses between the test and retest measurement results revealed no statistically significant difference (Table 5).

**Table 5:** DABBS-T test–retest results and mean scores (*n* = 30)

	Mean		Test values				ICC
	1. X ± SD	2. X ± SD	*r*	*p*	*t*	*p*	
F1	5.53 ± 1.63	5.70 ± 1.31	0.926	.000	− 1.409	0.169	0.950
F2	14.06 ± 6.72	13.33 ± 5.89	0.913	.000	1.459	0.155	0.950
F3	11.10 ± 3.48	11.03 ± 3.02	0.903		0.249	0.810	0.944
DABSS Total	30.70 ± 10.23	30.06 ± 8.44	0.947	.000	0.989	0.331	0.964

*F* Factor, *r* Pearson Correlation Coefficient, *t* Paired sample *t* test, *ICC* Intraclass Correlation Coefficient

^**^p: 0.01

## Discussion

Death anxiety is a multidimensional phenomenon that not only affects individuals’ religious and spiritual beliefs but also shapes their psychological well-being, lifestyle preferences, and health-related behaviors (Menzies et al., 2022; Zuccala et al., 2022). In this regard, death anxiety is closely associated with existential meaning-making processes that are often informed by religious and spiritual belief systems, positioning it within the broader religion–health framework. However, in Türkiye, there is no valid and reliable measurement tool that enables the assessment of individuals’ feelings, thoughts, and behaviors regarding death anxiety.

### Validity

I-CVI and S-CVI are methods used to assess content validity. In the literature, the threshold value for the I-CVI is 0.78, while this value is accepted as 0.90 for the S-CVI. Results above these values indicate strong content validity (Polit & Beck, 2008). Therefore, the Turkish form of the DABBS was found to have sufficient content validity (Polit & Beck, 2008; Polit et al., 2007).

In this study, firstly exploratory and then confirmatory factor analyses were conducted to determine the construct validity of the Turkish version of DABBS. A KMO value above 0.80 indicates that the sample is sufficient (Kaiser, 1974; Tabachnick et al., 2019). In this study, the KMO value was determined to be above 0.80. Therefore, the sample size was determined to be sufficient.

The significance of the Bartlett Test of Sphericity calculated in this study explains the presence of significant correlations between the variables (Shrestha, 2021; Williams et al., 2010). These results showed that the data set was suitable for confirmatory factor analysis.

According to the CFA results in this study, the factor loadings weighted for each item according to the three-factor structure varied between 0.530 and 0.940. In the literature, it is recommended to remove items with factor loadings below 0.30; however, because all items in this study exceeded 0.30, no items were removed (Büyüköztürk, 2007; Taljaard et al., 2018).

The CFA results supported the consistency of the scale with its original structure. The fit indices obtained in this study (*χ*^2^/SD = 2.00, RMSEA = 0.072) were evaluated in terms of model adequacy. The item with the highest arithmetic mean is 9. It concerns the sudden loss of a loved one. The fact that participants scored higher on this item than on statements about their own deaths may be a Türkiye-specific phenomenon.

In the literature, if the RMSA value is equal to or less than 0.05, it indicates a perfect fit (Seçer, 2020; Shadfar & Malekmohammadi, 2013; Woo, 2017). The fit indices obtained in this study fell within these acceptable limits. Similar results were obtained in the validity and reliability study of the Chinese version of the DABBS by Dong et al., which supports the findings of this study.

This finding supports that the Turkish form of the DABBS possesses a valid factor structure consistent with the original scale, comprising three subscales. Mazidi et al. found similar findings in the Persian version in their study of adolescents. These findings may also indicate that death is universal and that there are commonalities as a concept.

### Reliabilty

Cronbach’s *α* coefficient was used to determine the internal consistency and reliability of the DABBS. Cronbach’s *α* values were calculated as 0.932 for the “Emotion” subscale, 0.861 for the “Beliefs” subscale, 0.849 for the “Behaviors” subscale, and 0.918 for the total DABBS scale.

The item–total correlation coefficients of the DABBS were found to range from 0.462 to 0.738. No item was removed from the DABBS-T based on these results. In the literature, a Cronbach’s *α* coefficient value between 0.80 and 1.00 is considered an indicator of high reliability (Pallant, 2020). In the original development study of the DABBS by Menzies and colleagues (2022), the Cronbach’s α value and McDonald’s Omega value for the total DABBS-T were found to be 0.90. These results indicate that the findings of the Turkish validity and reliability study of the DABBS are consistent with the findings of the original scale.

To determine the stability of the scale over time, a test–retest analysis was conducted two weeks after the study, and the correlation coefficients results indicated a strong, positive, and statistically significant correlation for the DABBS-T. In this study, intraclass correlation coefficients (ICC) were calculated to support reliability, and these values were found to range between 0.944 and 0.964. In the literature, an ICC value above 0.90 indicates excellent reliability (Koo & Li, 2016). These findings indicate that the DABBS-T provides a high level of stability in repeated administrations. These results also demonstrate a high level of understanding of death-related anxieties in Türkiye. A Turkish proverb says that fear cannot prevent death. Because death is universal and a major source of anxiety in every culture, it was thought that DABBS-T would be compatible in terms of content in other cultures as well as in Türkiye.

Although death is known to be an inevitable reality, individuals within the same society may respond to this reality differently. DABBS-T will be particularly helpful in clinics with high mortality rates, guiding patients and their families in assessing their spiritual needs, understanding their beliefs, behaviors, and emotions related to death, and providing supportive care. Furthermore, assessing the anxieties related to death of all individuals outside of hospital settings will be a valuable tool for evaluating health behaviors and understanding the meaning of life.

## Limitation of the Study

This study has several limitations. First, the data were collected within a limited time frame and from participants who were reached during this period. Therefore, the findings may not be fully generalizable to the entire population. Second, the study relied on self-reported data, which may be influenced by participants’ subjective perceptions and response biases. Third, the absence of parallel forms is another limitation of the study. In addition, EFA was not conducted, and the sample was not divided into independent subsamples for exploratory and confirmatory analyses. This may limit the robustness of the factor structure evaluation and should be addressed in future validation studies.

## Conclusion

In this study, the Turkish version of the DABBS, comprising 18 items and three subscales, was demonstrated to be a valid, reliable, and practical tool for evaluating individuals’ emotions, beliefs, and behaviors related to death anxiety. Given that death anxiety directly affects health-related lifestyles and behaviors, the scale may serve as a valuable instrument for identifying its associated components and for guiding the design of appropriate intervention studies.

Furthermore, since beliefs and avoidance behaviors are often deeply intertwined with religious and spiritual frameworks, this scale provides a clinical foundation for understanding how spiritual convictions influence death-related attitudes. Moreover, as a culturally adapted measure, the scale holds considerable potential to advance both research and clinical practice by enabling the comprehensive assessment of death anxiety-related emotions, beliefs, and behaviors among individuals in Türkiye. Ultimately, the use of the DABBS-T in healthcare settings can facilitate the development of holistic and culturally sensitive interventions that integrate both psychological support and spiritual care for individuals facing existential concerns.

Therefore, the availability of culturally validated instruments assessing death-related cognitions may contribute to future research exploring the intersection of religion, spirituality, and health within diverse populations.

## Data Availability

The data supporting this study’s findings are available from the corresponding author upon reasonable request.

## The Death Anxiety Beliefs and Behaviors Scale (DABBS)

Ölüm Kaygısı İnançları ve Davranışları Ölçeği (ÖKİDÖ).

**Table:**

	Kesinlikle kabul etmiyorum 1	Kabul etmiyorum 2	Kararsızım 3	Kabul ediyorum 4	Kesinlikle kabul ediyorum 5
1. Ölüm konusunda kaygılı hissediyorum					
2. Bir gün ölecek olmam gerçeği korkutuyor					
3. Ölmekten korkuyorum					
4. Ölüm beni korkutuyor					
Aşağıda, deneyimleyebileceğiniz ölümle ilgili düşünce, inanç ve tutumların bir listesi bulunmaktadır. Lütfen her bir düşüncenin sizi ne sıklıkta rahatsız ettiğini 1’den başlayarak derecelendirerek belirtiniz 1 (‘hiç düşünmem’) ila 5 (‘her zaman düşünürüm’)					
	Hiç düşünmem	Nadiren düşünürüm	Bazen dünürüm	Çoğu zaman düşünürüm	Her zaman düşünürüm
5. İstediğim her şeyi deneyimlemek için zamanımın olmaması korkunç olurdu					
6. Yalnız ölmek korkunç olurdu					
7. Ölümüm acı verici bir deneyim olacak					
8.Sevdiklerim olmadan yaşlanmayla baş edemezdim					
9 Aniden sevdiğim birini kaybedersem bu durum beni mahveder					
10. Ölüm döşeğimde, ölümü gerektiği kadar cesurca karşılayamayacağım					
11. Önemsediğim birinin ölümcül bir hastalığa yakalanmasıyla başa edemezdim					
Aşağıda, bazı kişilerin kaçınabileceği etkinliklerin bir listesi bulunmaktadır. Lütfen bu durumlardan her birinden ne sıklıkla kaçınacağınızı 1’den ("Asla kaçınmam") 5’e kadar (“Her zaman kaçınırım”). belirtin					
	Asla kaçınmam	Nadiren kaçınırım	Bazen kaçınırım	Çoğu zaman kaçınırım	Her zaman kaçınırım
12. Ölümle ilgili medya hikayelerini izlemek veya okumak					
13. Ölümcül bir hastalık teşhisi aldığımı düşünmek					
14. İçinde ölmek üzere olan bir karakter olan roman okumak					
15. Sevdiğim birisinin öldüğünü düşünmek					
16. İçinde ölmek üzere olan bir karakter bulunan bir film ya da TV programı izlemek					
17. Öldüğümü düşünmek					
18. Ölümcül hastalık teşhisi konan birinin anılarını veya makalelerini okumak					
